# Multimodal prevention of emergence cough following nasal endoscopic surgery under general anesthesia: a double-blind randomized trial

**DOI:** 10.3389/fmed.2024.1288978

**Published:** 2024-01-24

**Authors:** Jing Xu, Pei Sun, Jia-Hui Ma, Dong-Xin Wang

**Affiliations:** ^1^Department of Anesthesiology, Peking University First Hospital, Beijing, China; ^2^Outcomes Research Consortium, Cleveland, OH, United States

**Keywords:** cough, extubation, emergence, ropivacaine, dexmedetomidine, remifentanil

## Abstract

**Purpose:**

Cough during emergence from anesthesia is a common problem and may cause adverse events. Monotherapy faces uncertainty in preventing emergence cough due to individual differences. We aimed to evaluate the efficacy and safety of multimodal intervention for preventing emergence cough in patients following nasal endoscopic surgery.

**Methods:**

In this double-blind randomized trial, 150 adult patients undergoing nasal endoscopic surgery were randomly allocated into three groups. For the control group (*n* = 50), anesthesia was performed according to clinical routine, no intervention was provided. For the double intervention group (*n* = 50), normal saline 3 mL was sprayed endotracheally before intubation, 0.4 μg/kg dexmedetomidine was infused over 10 min after intubation, and target-controlled remifentanil infusion was maintained at an effect-site concentration of 1.5 ng/mL before extubation after surgery. For the multimodal intervention group (*n* = 50), 0.5% ropivacaine 3 mL was sprayed endotracheally before intubation, dexmedetomidine and remifentanil were administered as those in the double intervention group. The primary endpoint was the incidence of emergence cough, defined as single cough or more from end of surgery to 5 min after extubation.

**Results:**

The incidences of emergence cough were 98% (49/50) in the control group, 90% (45/50) in the double group, and 70% (35/50) in the multimodal group, respectively. The incidence was significantly lower in the multimodal group than those in the control (relative risk 0.71; 95% CI 0.59 to 0.86; *p* < 0.001) and double (relative risk 0.78; 95% CI 0.63 to 0.95; *p* = 0.012) groups; the difference between the double and control groups was not statistically significant (relative risk 0.92; 95% CI 0.83 to 1.02; *p* = 0.20). The severity of sore throat was significantly lower in the multimodal group than that in the control group (median difference-1; 95% CI −2 to 0; *p* = 0.016). Adverse events did not differ among the three groups.

**Conclusion:**

For adult patients undergoing endonasal surgery, multimodal intervention including ropivacaine topical anesthesia before intubation, dexmedetomidine administration after intubation, and remifentanil infusion before extubation after surgery significantly reduced emergence cough and was safe.

## Introduction

Smooth recovery from general anesthesia is a matter of concern to anesthesiologists. A key element is to avoid coughing during emergence (1), of which the reported rate is up to 91% (2–4). Emergence cough may cause hemodynamic changes and laryngospasm, and increase intraocular and intracranial pressures (1). In specific conditions, it is even associated with negative pressure pulmonary edema and sternal dehiscence (5, 6). During the Covid-19 pandemic, cough was considered to be a high-risk aerosol-generating activity (7); much attention was paid to avoid coughing during extubation in order to protect healthcare providers from inhaling aerosols or contacting droplets.

Several methods have been reported effective in relieving emergence cough, including topical anesthesia (8), dexmedetomidine administration (3), intravenous lidocaine (9), and remifentanil infusion (10). However, monotherapy faces uncertainty in clinical efficacy due to individual differences, whereas increasing dosage may lead to drug-related side effects such as local anesthetic toxicity, delayed emergence, bradycardia, and respiratory depression (9, 11). How to reduce emergence cough effectively and safely remains a problem to be solved.

In our center, endonasal surgery is usually performed under general anesthesia with endotracheal intubation. Patients undergoing endonasal surgery are relatively young and have a high rate of coughing during emergence from anesthesia. We supposed that a multimodal intervention including topical anesthesia, dexmedetomidine administration, and remifentanil infusion might be more effective in relieving emergence cough when compared with double intervention or no intervention. This randomized trial was designed to evaluate the efficacy and safety of a multimodal intervention in patients after endonasal surgery.

## Methods

### Study design

This was a double-blind, randomized, controlled trial with three parallel arms. The study protocol was approved by the Biomedical Research Ethics Committee of Peking University First Hospital (No.2021-015; issued on March 4, 2021) and registered with Chinese Clinical Trial Registry (chictr.org.cn; ChiCTR2100044573; registered on March 24, 2021). The trial was conducted in Peking University First Hospital (Beijing, China) and reported in accordance with the Consolidated Standards of Reporting Trials guidelines (12). Written informed consent was obtained from each participant.

### Participants

We enrolled patients aged 18 to 65 years who were scheduled to undergo elective endonasal surgery with an estimated duration between 30 and 180 min. We excluded those who met any of the following criteria: (1) anticipated difficult endotracheal intubation; (2) comorbid with respiratory disease or recent respiratory tract infection, or an American Society of Anesthesiologists (ASA) classification of III or higher; or (3) estimated to be at risk of reflux and aspiration.

### Randomization, intervention, and blinding

Random numbers were generated using the SAS software 9.3 with a block size of six in a 1:1:1 ratio and were sealed in sequentially numbered opaque envelopes. An anesthesiologist (JX) enrolled eligible patients. Shortly before anesthesia, a study nurse (GYG) who otherwise was not involved in the trial opened the envelopes according to the recruitment sequence and prepared the study drugs according to the randomization results. The envelops were then closed again until the end of the trial.

The study nurse prepared the following drugs: 3 mL of 0.5% ropivacaine (AstraZeneca AB, Sodertalje, Sweden) or placebo (normal saline) in an identical syringe which was connected to a laryngotracheal topical anesthesia kit (Tuoren Holding Group Co., Ltd., Xinxiang, China), remifentanil (Humanwell Healthcare Group Co., Ltd., Yichang, China) dissolved in normal saline to a concentration of 20 μg/mL, and dexmedetomidine (Yangtze River Pharmaceutical Group Co., Ltd., Taizhou, China) diluted with normal saline to a concentration of 10 μg/mL.

The anesthesiologist (JX) who performed anesthesia gave the prepared study drugs according to the randomization results. In this way, the enrolled patients were randomly allocated into three groups. For the control group, anesthesia was performed according to clinical routine, no intervention was provided. For the double intervention group, placebo (normal saline 3 mL) was sprayed endotracheally before intubation, 0.4 μg/kg dexmedetomidine was infused over 10 min after intubation, and target-controlled remifentanil infusion was maintained at an effect-site concentration of 1.5 ng/mL before extubation after surgery. For the multimodal intervention group, 0.5% ropivacaine 3 mL was sprayed endotracheally before intubation, 0.4 μg/kg dexmedetomidine was infused over 10 min after intubation, and target-controlled remifentanil infusion was maintained at an effect-site concentration of 1.5 ng/mL before extubation after surgery.

The responsible anesthesiologist was aware of the assignment to the control group, but not the assignment to the other two groups. At the end of surgery, the remifentanil infusion pump was covered by a piece of cloth to keep masking. Both patients and investigator for outcome assessment (PS) were blinded to study group assignment.

### Anesthesia and perioperative care

No pre-anesthesia medication was given. Intraoperative monitoring included electrocardiogram, noninvasive blood pressure, pulse oxygen saturation (SpO_2_), train-of-four (TOF) stimulation, bispectral index (BIS), end-tidal concentration of carbon dioxide, and inhalational anesthetic concentration.

Anesthesia was induced with propofol (1–2 mg/kg), sufentanil (0.2 μg/kg), remifentanil (effect-site target concentration 1–3 ng/mL), and rocuronium (0.6 mg/kg) or cis-atracurium (0.15 mg/kg). Endotracheal intubation was completed for all patients, using a polyvinyl chloride tracheal tube (I.D. 7.5 for male and I.D. 7.0 for female) lubricated with oxybuprocaine gel. Anesthesia was maintained with infusion of propofol and remifentanil and inhalation of sevoflurane, targeting a BIS value between 40 and 60. Additional cis-atracurium was administered when considered necessary. Mechanical ventilation was established (tidal volume 8–10 mL/kg, PEEP 3–5 cmH_2_O, and respiratory rate 12–14 times per minute) with an oxygen-air mixture (FiO_2_ 50%). Vasoactive drugs were used to maintain hemodynamics stable.

About 10 min before the end of surgery, propofol and sevoflurane were suspended; nonsteroidal anti-inflammatory drugs and ondansetron were administered. At the end of surgery, a mixture of neostigmine and atropine was used to antagonize residual muscle relaxation when indicated, as guided by TOF monitoring. Extubation was performed when patients regained consciousness and muscle strength, had stable hemodynamics, and had adequate gas exchange and airway protection. Patients were transferred to the post-anesthesia care unit (PACU) for at least 30 min before being sent back to the general wards. Other managements were provided per routine.

### Data collection and outcome assessment

Baseline data included demographic and morphometric characteristics, history of smoking, preoperative comorbidities and medications, American Society of Anesthesiologists (ASA) classification, and modified Mallampati grade. Intra-and postoperative data included duration of anesthesia (from anesthesia induction to propofol termination), types and doses of medications during anesthesia, and types and duration of surgery.

Our primary endpoint was the incidence of emergence cough. Anesthesia emergence was defined as the period from the end of surgery to 5 min after extubation; cough was defined as a sudden contraction of the abdominal muscle (13). The severity of cough was classified into four grades: grade 0, no cough; grade 1, single cough; grade 2, unsustained (<5 s) cough; grade 3, sustained (>5 s) cough or bucking (8, 13). Patients with cough of grade 1 or higher were noted as having emergence cough.

The secondary endpoints included incidence of moderate-to-severe emergence cough (grade 2 or higher), cough during PACU stay, variance of heart rate during emergence, Richmond Agitation-Sedation Scale [score ranges from −5 (unarousable) to +4 (combative) and 0 indicates alert and calm] (14) at 5 min and 20 min after extubation, and numeric rating scale (an 11-point scale where 0 = no pain and 10 = the worst pain) of sore throat before leaving the PACU, as well as incidences of emergence cough and moderate-to-severe emergence cough at different timepoints (pre-extubation, upon extubation, 5 min post-extubation, and overall). Cardiac acceleration was defined as an increase of heart rate >20% from baseline (average ward value) during anesthesia emergence. Cardiac deceleration was defined as a decrease of heart rate >20% from baseline during anesthesia emergence.

Other endpoints included time to eye opening (from propofol termination to eye opening), time to extubation (from end of surgery to extubation), memory of extubation (defined as patients being able to recall the course of tracheal extubation), length of PACU stay, length of hospital stay after surgery, and occurrence of postoperative complications.

Adverse events were monitored for up to 72 h after surgery. Specifically, we monitored the occurrence of bradycardia (heart rate <50 beat/min or a decrease of >30% from baseline, and required therapy), tachycardia (heart rate >100 beat/min or an increase of >30% from baseline, and required therapy), hypotension (systolic blood pressure <90 mmHg or a decrease of >30% from baseline, and required therapy), hypertension (systolic blood pressure >180 mmHg or an increase of >30% from baseline, and required therapy), delayed recovery (time to extubation >30 min), desaturation (SpO_2_ <90% in room air), respiratory depression (respiratory rate <10 breaths/min), epistaxis (newly occurred nasal bleeding after surgery that required therapeutic intervention), and postoperative nausea and vomiting (development of any nausea, retching, or vomiting).

### Statistical analysis

#### Sample size estimation

According to previous studies, the incidences of emergence cough ranged from 66% to 91% in the absence of specific interventions (4, 15, 16), from 32% to 81% with remifentanil infused at different target (2, 4, 10), and from 26% to 33% after lidocaine topical anesthesia (8, 17). A study reported an emergence cough incidence of 35% after airway spraying of 0.75% ropivacaine (18). We assumed that cough incidences would be 90%, 70%, and 55% in the control group, double intervention group, and multimodal intervention group, respectively. With the significance level set at 0.05/3 = 0.0167 and power at 80%, the calculated sample size required to detect differences among the three groups was 42 patients in each group. Considering a drop-out rate of about 20%, we planned to enroll 50 patients in each group. Sample size calculation was performed using PASS 11.0 software (NCSS Statistical Software, Utah, United States).

#### Data analysis

Data analysis was performed in the intention-to-treat population. For the primary outcome, analysis was also performed in the per-protocol population, in which case patients with protocol deviation were excluded. For intraoperative data, quantitative data were compared with Kruskal–Wallis test; qualitative data were compared with chi-squared test or Fisher’s exact test.

Our primary endpoint, the incidence of emergence cough, was compared with chi-squared test with differences between groups manifested as relative risks (RRs) and two-sided 95% confidence intervals (CIs). For secondary endpoints, quantitative data were compared with Kruskal–Wallis test; qualitative data were compared with chi-squared test or Fisher’s exact test. The differences between groups were expressed as RRs or median differences (MDs) and 95% CIs. As exploratory analyses, we also compared the incidences of emergence cough and moderate-to-severe emergence cough of non-smokers and smokers among three groups.

A two-side *p* < 0.05 was considered statistically significant. For multiple comparisons, the threshold of significance was adjusted using Bonferroni method; *p* < 0.017 (0.05/3) was considered statistically significant. Statistical analysis was performed with the SPSS 21.0 software package (IBM SPSS Inc., Chicago, IL, United States).

## Results

### Patient recruitment and characteristics

**Figure 1:**
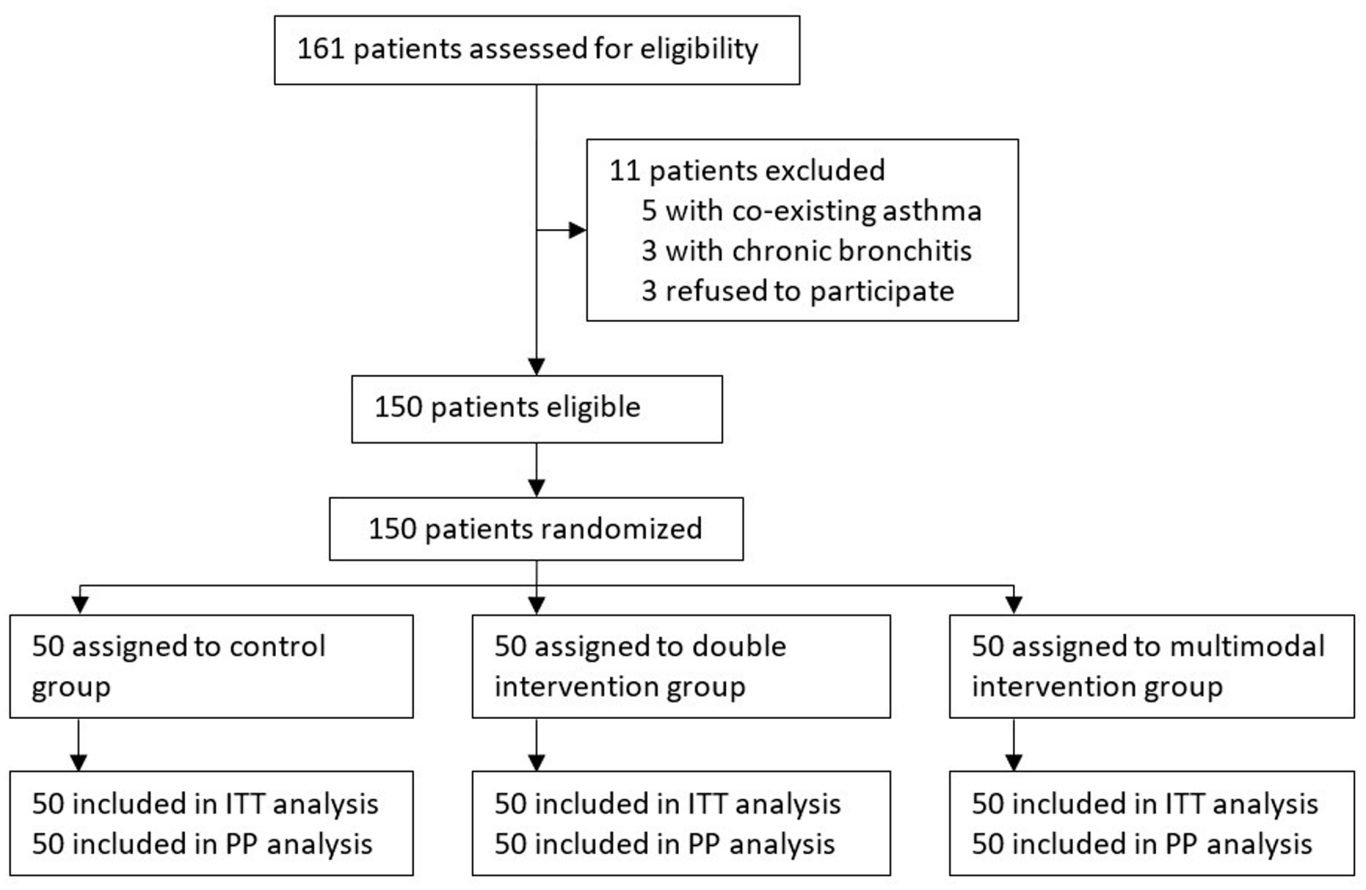
Flowchart of the trial. ITT, intention-to-treat; PP, per-protocol.

From March 30, 2021 to September 30, 2022, 470 patients underwent intranasal surgery under general anesthesia. Among these, 161 patients were assessed for eligibility; 150 patients were recruited and randomized into three groups, with 50 patients in each group. No protocol deviation occurred during the study period. All enrolled patients were included in the intention-to-treat and per-protocol analyses (Figure 1).

Of the enrolled patients, the mean age was 45 years, 65% (98/150) were male, 57% (85/150) were non-smokers, 53% (80/150) had no comorbidity, and 26% (39/150) were expected to have difficult airway (Mallampati grade III or IV). Baseline data were balanced among the three groups (Table 1).

**Table 1:** Baseline data.

	Control (*N* = 50)	Double intervention (*N* = 50)	Multimodal intervention (*N* = 50)
Age (year)	44 (13)	48 (12)	43 (12)
Male	34 (68%)	32 (64%)	32 (64%)
Body mass index (kg m^−2^)	25.9 (4.0)	24.9 (3.3)	26.0 (4.3)
**History of smoking**			
Never	26 (52%)	29 (58%)	30 (60%)
Previous smoking^a^	8 (16%)	12 (24%)	6 (12%)
Current smoking^b^	16 (32%)	9 (18%)	14 (28%)
**Preoperative comorbidities**			
Hypertension	11 (22%)	11 (22%)	15 (30%)
Diabetes mellitus	4 (8%)	2 (4%)	3 (6%)
Stroke	1 (2%)	0 (0%)	1 (2%)
OSAS	5 (10%)	3 (6%)	8 (16%)
Other diseases^c^	6 (12%)	6 (12%)	10 (20%)
**Number of comorbidities**			
None	30 (60%)	29 (58%)	21 (42%)
1	14 (28%)	19 (38%)	23 (46%)
≥2	6 (12%)	2 (4%)	6 (12%)
**Preoperative medications**			
ACEI^d^	2 (4%)	0 (0%)	0 (0%)
Antihypertensive drug	9 (18%)	10 (20%)	14 (28%)
Glucocorticoids^e^	1 (2%)	1 (2%)	2 (4%)
**ASA classification**			
I	9 (18%)	14 (28%)	13 (26%)
II	41 (82%)	36 (72%)	37 (74%)
**Modified Mallampati score** ^f^			
I	7 (14%)	7 (14%)	10 (20%)
II	30 (60%)	33 (66%)	24 (48%)
III	11 (22%)	9 (18%)	15 (30%)
IV	2 (4%)	1 (2%)	1 (2%)

Data are mean (standard deviation) or *n* (%). OSAS, obstructive sleep apnea syndrome; ACEI, angiotensin-converting enzyme inhibitors; ASA, American Society of Anesthesiologists.

a Stop smoking for at least 2 weeks.

b Smoking even a single cigarette within the last 2 weeks.

c Including coronary disease, chronic kidney disease and chronic gastritis.

d Taking ACEI every day for at least 1 month.

e Taking inhaled or oral corticosteroids within the last week.

f Class I: the soft palate, entire uvula, fauces, and pillars are visible; class II: the soft palate, majority of uvula, and fauces are visible; class III: the soft palate and base of uvula are visible; class IV: only the hard palate is visible.

More than half of our patients [56% (84/150)] underwent functional endoscopic sinus surgery. Intraoperative data including medications during anesthesia, type of surgery, and durations of anesthesia and surgery were comparable among the three groups (Table 2).

**Table 2:** Intraoperative data.

	Control (*N* = 50)	Double intervention (*N* = 50)	Multimodal intervention (*N* = 50)	*p*-value
Anesthesia duration (min)	103 [81, 133]	98 [72, 129]	108 [87, 163]	0.16^d^
**Medications during anesthesia**				
Propofol (mg)	400 [307, 559]	355 [248, 498]	427 [306, 582]	0.11^d^
Sufentanil (μg)	15 [12, 18]	15 [12, 15]	15 [13, 18]	0.42^d^
Use of rocuronium	49 (98%)	48 (96%)	48 (96%)	>0.99^f^
Rocuronium (mg)^a^	50 [40, 50]	48 [40, 50]	50 [40, 50]	0.78^d^
Additional cis-atracurium	3 (6%)	1 (2%)	6 (12%)	0.16^f^
Use of ephedrine	24 (48%)	27 (54%)	27 (54%)	0.79^e^
Ephedrine (mg)^a^	0 [0, 6.0]	4.5 [0, 6.0]	3.0 [0, 6.0]	0.67^d^
Use of metaraminol	21 (42%)	26 (52%)	24 (48%)	0.60^e^
Metaraminol (μg)^a^	0 [0, 625]	250 [0, 1,000]	0 [0, 1,000]	0.67^d^
Use of neostigmine	29 (58%)	28 (56%)	30 (60%)	0.92^e^
Neostigmine (mg)^a^	1 [0, 2]	1 [0, 2]	1.5 [0, 2]	0.89^d^
Site of surgery				0.32^f^
Nasal septum	10 (20%)	11 (22%)	13 (26%)	
Functional endoscopic sinus	29 (58%)	31 (62%)	24 (48%)	
Combined^b^	7 (14%)	1 (2%)	5 (10%)	
Others^c^	4 (8%)	7 (14%)	8 (16%)	
Surgery duration (min)	87 [72, 126]	88 [59, 114]	95 [75, 150]	0.24^d^

Data are median (interquartile range) or *n* (%).

a Among patients who were given the medications.

b Functional endoscopic sinus surgery and nasal septum surgery.

c Including nasal tumor resection and rhinodacryocystostomy.

d Kruskal–Wallis test.

e Chi-squared test.

f Fisher’s exact test.

### Efficacy outcomes

The incidences of emergence cough were 98% (49/50) in the control group, 90% (45/50) in the double intervention group, and 70% (35/50) in the multimodal intervention group, respectively. The incidence was significantly lower in the multimodal group than those in the control group (RR 0.71; 95% CI 0.59 to 0.86; *p* < 0.001) and the double intervention group (RR 0.78; 95% CI 0.63 to 0.95; *p* = 0.012); the difference between the double intervention and control groups was not statistically significant (RR 0.92; 95% CI 0.83 to 1.02; *p* = 0.20; Table 3).

**Table 3:** Postoperative outcomes.

	Control (*N* = 50)	Double intervention (*N* = 50)	Multimodal intervention (*N* = 50)	Multimodal vs. control		Multimodal vs. double		Double vs. control	
				RR/MD (95% CI)	*p*-value^a^	RR/MD (95% CI)	*p*-value^a^	RR/MD (95% CI)	*p*-value^a^
**Primary outcome**									
Emergence cough^b^	49 (98%)	45 (90%)	35 (70%)	0.71 (0.59, 0.86)	**<0.001** ^j^	0.78 (0.63, 0.95)	**0.01** ^j^	0.92 (0.83, 1.02)	0.20^j^
**Secondary outcomes**									
Moderate-to-severe cough^c^	43 (86%)	43 (86%)	24 (48%)	0.56 (0.41, 0.76)	**<0.001** ^j^	0.56 (0.41, 0.76)	**<0.001** ^j^	1.00 (0.85, 1.17)	>0.99^j^
Cough during PACU stay	8 (16%)	7 (14%)	5 (10%)	0.63 (0.22, 1.78)	0.37^j^	0.71 (0.24, 2.10)	0.54^j^	0.88 (0.34, 2.23)	0.78^j^
Cardiac acceleration^d^	16 (32%)	8 (16%)	8 (16%)	0.50 (0.24, 1.06)	0.06^j^	1.00 (0.41, 2.46)	>0.99^j^	0.50 (0.24, 1.06)	0.06^j^
Cardiac deceleration^e^	5 (10%)	10 (20%)	10 (20%)	2.00 (0.74, 5.43)	0.16^j^	1.00 (0.46, 2.19)	>0.99^j^	2.00 (0.74, 5.43)	0.16^j^
Memory of extubation	2 (4%)	3 (6%)	2 (4%)	1.00 (0.15, 6.82)	>0.99^k^	0.67 (0.12, 3.82)	>0.99^k^	1.50 (0.26, 8.60)	>0.99^k^
Postoperative complications	5 (10%)	3 (6%)	6 (12%)	1.20 (0.39, 3.68)	0.75^k^	2.00 (0.53, 7.56)	0.49^k^	0.60 (0.15, 2.38)	0.72^k^
Time to eyes opening (min)	9 [6, 13]	11 [7, 14]	10 [7, 12]	MD = 0 (−2, 2)	0.88^i^	MD = −1 (−3, 1)	0.25^i^	MD = 1 (−1, 3)	0.29^i^
Time to extubation (min)	6 [4, 9]	7 [4, 9]	7 [4, 10]	MD = 1 (−1, 2)	0.29^i^	MD = 0 (−1, 2)	0.52^i^	MD = 0 (−1, 2)	0.71^i^
Intensity of sore throat (point)^f^	2 [1, 4]	2 [0, 3]	1 [0, 2]	MD = −1 (−2, 0)	**0.016** ^i^	MD = 0 (−1, 0)	0.23^i^	MD = 0 (−1, 0)	0.26^i^
Length of PACU stay (min)	33 [31, 37]	33 [30, 37]	33 [28, 37]	MD = −1 (−3, 1)	0.47^i^	MD = −1 (−3, 1)	0.51^i^	MD = 0 (−2, 2)	0.89^i^
Length of hospital stay (day)	4 [3, 4]	3 [3, 4]	3 [3, 4]	MD = 0 (0, 1)	0.97^i^	MD = 0 (0, 1)	0.32^i^	MD = 0 (−1, 0)	0.38^i^
**Exploratory analyses**									
*Emergence cough* ^b^									
Non-smokers^g^	26/26 (100%)	26/29 (90%)	16/30 (53%)	0.53 (0.38, 0.75)	**<0.001** ^j^	0.60 (0.42, 0.85)	**0.002** ^j^	0.90 (0.79, 1.02)	0.24^k^
Smokers^h^	23/24 (96%)	19/21 (91%)	19/20 (95%)	0.99 (0.87, 1.13)	>0.99^k^	1.05 (0.89, 1.25)	>0.99^k^	0.94 (0.80, 1.11)	0.59^k^
*Moderate-to-severe cough* ^c^									
Non-smokers^g^	24/26 (92%)	26/29 (90%)	10/30 (33%)	0.36 (0.22, 0.61)	**<0.001** ^j^	0.37 (0.22, 0.63)	**<0.001** ^j^	0.97 (0.82, 1.15)	>0.99^k^
Smokers^h^	19/24 (79%)	17/21 (81%)	14/20 (70%)	0.88 (0.62, 1.26)	0.48^i^	0.87 (0.61, 1.23)	0.48^k^	1.02 (0.76, 1.37)	>0.99^k^

Data are *n* (%) or median (interquartile range). PACU, post-anesthesia care unit; RR, relative risk; MD, median difference; CI, confidence interval. *p*-values in bold indicate <0.017.

a *p* < 0.017 as significance threshold after Bonferroni correction.

b Occurrence of cough of grade 1 or higher during the emergence period (from the end of surgery to 5 min after extubation). The severity of cough was classified into four grades: grade 0, no cough; grade 1, single cough; grade 2, unsustained (<5 s) cough; grade 3, sustained (>5 s) cough or bucking.

c Occurrence of cough of grade 2 or higher during the emergence period.

d Increase of heart rate >20% from baseline (average ward value) during the emergence period.

e Decrease of heart rate >20% from baseline (average ward value) during the emergence period.

f Assessed with an 11-point scale where 0 = no pain and 10 = the worst pain.

g Patients with no smoking history.

h Patients who currently smoked or with a smoking history.

i Kruskal–Wallis test.

j Chi-squared test.

k Fisher’s exact test.

**Figure 2:**
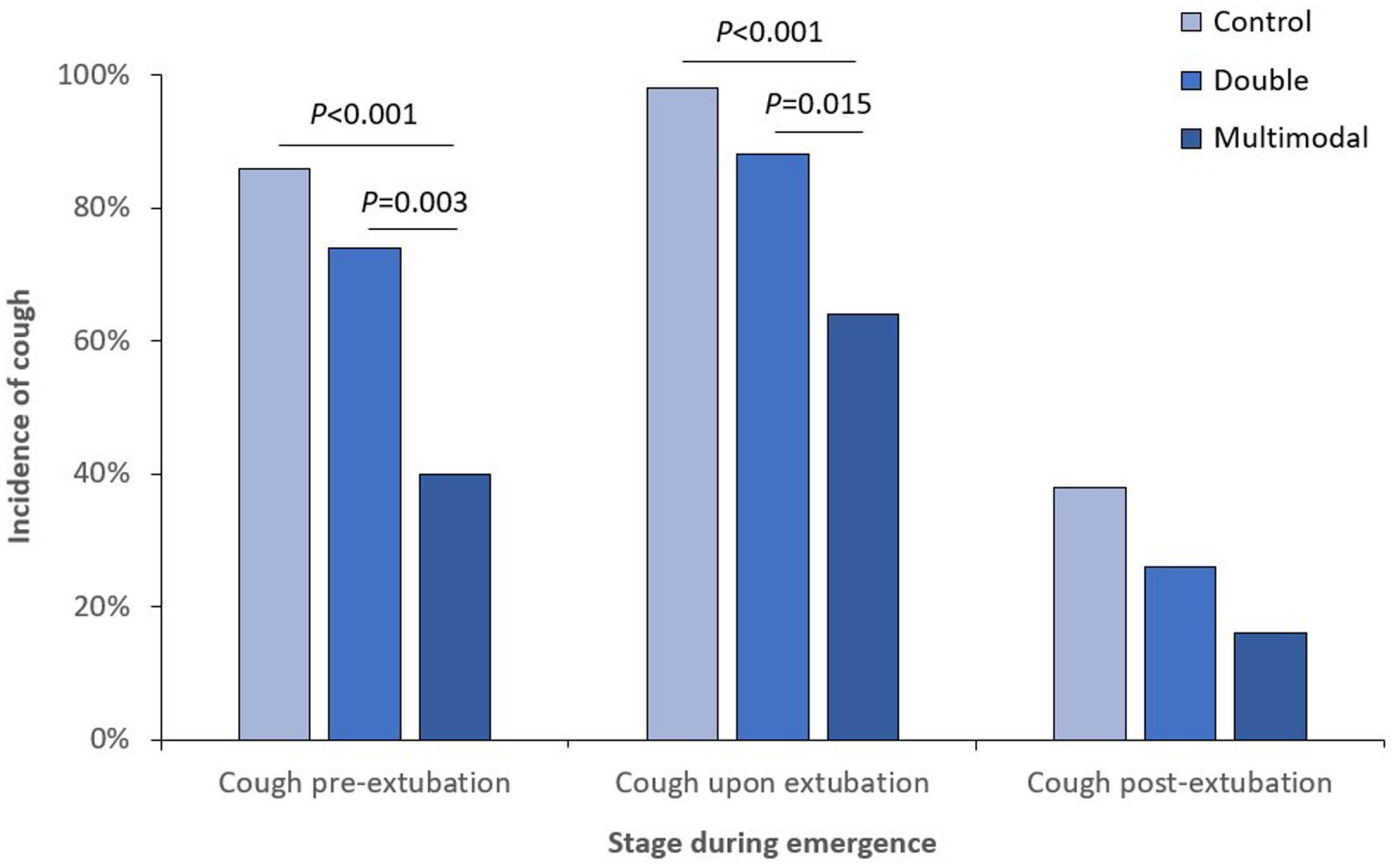
Incidence of cough at different stages during anesthesia emergence.

**Figure 3:**
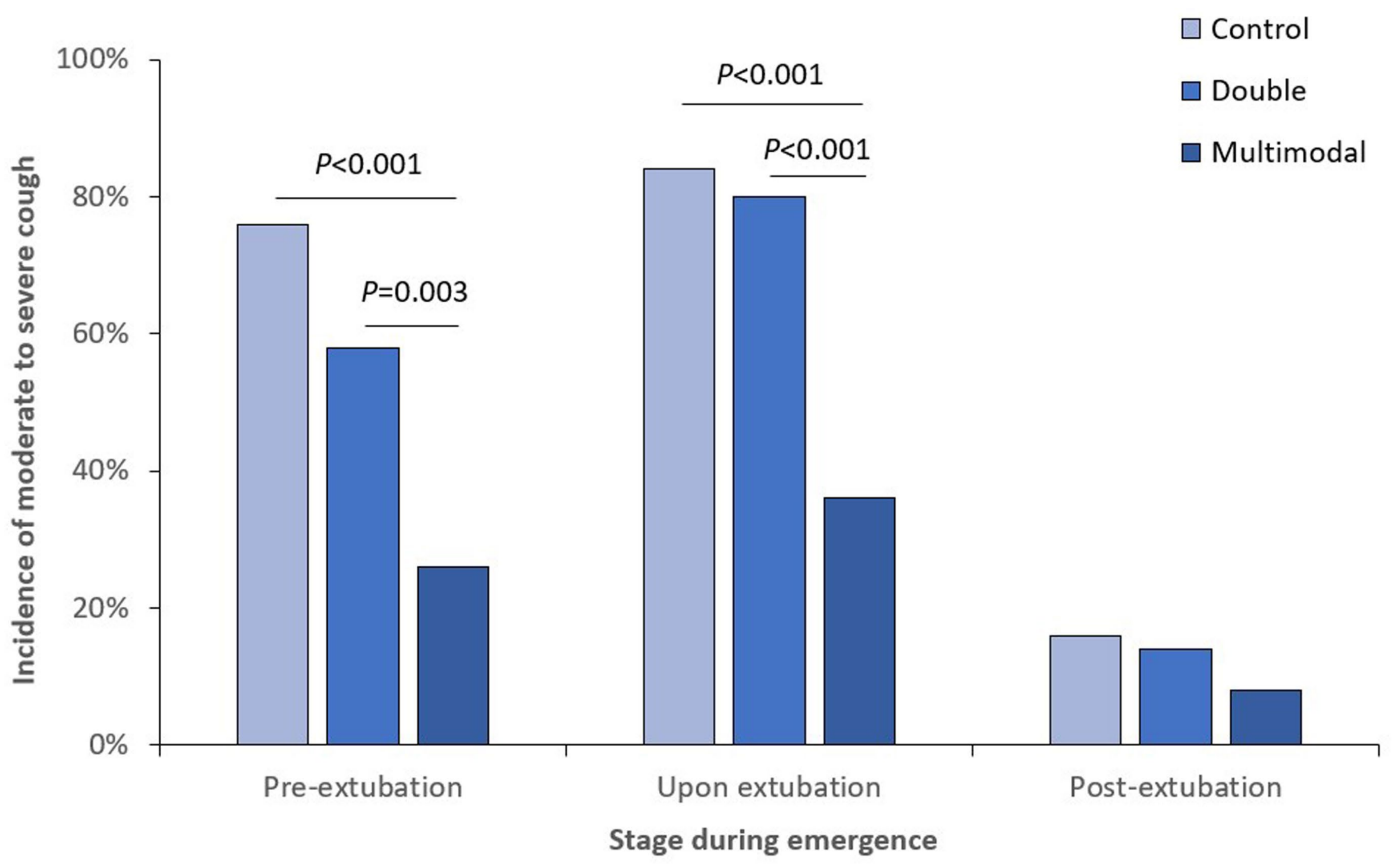
Incidence of moderate-to-severe cough at different stages during anesthesia emergence.

When looking at different stages of anesthesia emergence, the incidence of cough was significantly lower in the multimodal group than those in the control and double groups at both pre-extubation and upon extubation stages (Figure 2); the incidence of moderate-to-severe cough was also significantly lower in the multimodal group than those in the control and double groups at both pre-extubation and upon extubation stages (Figure 3). The grade of cough was significantly lower in the multimodal group than that in the control group at pre-extubation, upon extubation, and post-extubation stages; the grade was significantly lower in the multimodal group than that in the double group at pre-extubation and upon extubation stages, and was significantly lower in the double group than that in the control group at pre-extubation stage (Supplementary Table S1).

Among secondary endpoints, the incidence of moderate-to-severe cough was significantly lower in the multimodal group than those in the control group (RR 0.56; 95% CI 0.41 to 0.76; *p* < 0.001) and the double group (RR 0.56; 95% CI 0.41 to 0.76; *p* < 0.001). The intensity of sore throat was significantly lower in the multimodal group than that in the control group (median difference − 1; 95% CI -2 to 0; *p* = 0.016). Other endpoints, including the incidence of cardiac acceleration during the emergence period, did not differ significantly among the three groups (Table 3).

As exploratory analyses in the subgroup of non-smokers, the incidences of emergence cough and moderate-to-severe cough were both lower in the multimodal group than those in the control and double groups. Whereas, in the subgroup of smokers, the incidences of emergence cough and moderate-to-severe cough did not differ among three groups (Table 3).

### Safety outcomes

Five patients (10%) in the double group developed postoperative nausea and vomiting, but the difference was not statistically significant when compared with either the control group or the multimodal group. Other adverse events did not differ among the three groups. No patient developed delayed emergence, hypotension, desaturation, or respiratory depression during the study period (Table 4).

**Table 4:** Safety outcomes.

	Control (*N* = 50)	Double intervention (*N* = 50)	Multimodal intervention (*N* = 50)	*p*-value
Bradycardia^a^	0 (0%)	2 (4%)	4 (8%)	0.17^h^
Tachycardia^b^	12 (24%)	8 (16%)	5 (10%)	0.17^g^
Hypertension^c^	1 (2%)	0 (0%)	0 (0%)	>0.99^h^
Hypotension^d^	0 (0%)	0 (0%)	0 (0%)	—
Delayed recovery^d^	0 (0%)	0 (0%)	0 (0%)	—
Desaturation^d^	0 (0%)	0 (0%)	0 (0%)	—
Respiratory depression^d^	0 (0%)	0 (0%)	0 (0%)	—
Epistaxis^e^	4 (8%)	2 (4%)	5 (10%)	0.63^h^
Nausea/vomiting^f^	0 (0%)	5 (10%)	1 (2%)	**0.048** ^h^

Data are *n* (%). *p*-values in bold indicate <0.05.

a Heart rate <50 beats/min or a decrease of >30% from baseline (average value in the ward).

b Heart rate >100 beats/min or an increase of >30% from baseline (average value in the ward).

c Systolic blood pressure >180 mm Hg or an increase of >30% from baseline (average value in the ward).

d No patient developed hypotension, delayed recovery, desaturation, or respiratory depression during the study period.

e Newly developed nasal bleeding after surgery that therapeutic intervention.

f Defined as the development of any nausea, retching, or vomiting after surgery.

g Chi-squared test.

h Fisher’s exact test.

## Discussion

Our results showed that the incidence of emergence cough remained high in adult patients following endonasal surgery. When compared with control patients (no intervention), multimode intervention including ropivacaine topical anesthesia, dexmedetomidine administration, and remifentanil infusion significantly reduced emergence cough, whereas double intervention including only dexmedetomidine administration and remifentanil infusion did not. The effect of multimodal intervention in preventing emergence cough seems more prominent in non-smokers.

According to previous surveys, the majority of anesthesiologists prefer to extubate patients after full awake, in order to reduce airway related complications (19, 20). However, awake extubation is associated with emergence cough which may also cause unfavorable outcomes or even severe complications (1, 5, 6). Smooth extubation can be achieved with some medications (3, 8–10, 21). In a network meta-analysis, dexmedetomidine has the highest rank in suppressing emergence cough, followed by remifentanil and others (22). But no single intervention is completely effective.

In a small sample size trial, a single-dose dexmedetomidine augmented the cough-suppressing effect of remifentanil infusion (23). We therefore adopted the dexmedetomidine-remifentanil combination in our double intervention group patients. However, we did not find clinically important cough-suppressing effects of the combined therapy. This could be attributed to the low-dose regimen in our patients. For example, in the study of Kim et al. (23), the effect-site concentrations of remifentanil for preventing cough in 50% and 95% of patients following nasal surgery were 2.15 ng/mL and 2.75 ng/mL, respectively, in the presence of 0.5 μg/kg dexmedetomidine. Whereas in our patients with double intervention, 0.4 μg/kg dexmedetomidine was administered after intubation, and remifentanil infusion was maintained at an effect-site concentration of 1.5 ng/mL after surgery. In accord with this, the time intervals to eye opening and extubation were similar among the three groups of our patients, indicating a relatively low-dose combination (24). Nevertheless, cough severity score was significantly lower in the double intervention group than in the control group at the pre-extubation stage, indicating some effects (Supplementary Table S1).

Lidocaine topicalization of the airway is also an effective way to relieve emergence cough (22). In the study of Minogue et al. (8), endotracheal spraying with 4% lidocaine at the time of intubation decreased the incidence of coughing before (26% vs. 66%, *p* < 0.01) and after (4% vs. 30%, *p* = 0.02) extubation. Other studies confirmed that both intravenous and intracuff lidocaine relieved emergence cough (9, 25), but were less effective than intratracheal administration (17, 26). However, 4% lidocaine for topical anesthesia is not commonly available in China. Whereas the effect of 2% lidocaine is not durable when used for topical anesthesia. For example, intratracheal administration of 2% lidocaine at a dose of 1 mg/kg did not prevent emergence cough when given 20–30 min before extubation (27).

Ropivacaine is an amide-type local anesthetic with slow onset and long duration of action. It is also used for topical eye anesthesia. In cataract surgery, 1% ropivacaine performs equally well as 4% lidocaine in efficacy and safety and provides sufficient and long-lasting topical analgesia (28); in pterygium surgery, 1% ropivacaine is effective and permits the whole procedure (29). When used for airway topical anesthesia by inhalation, 1% ropivacaine and 4% lidocaine provides similar efficiency and duration (30). In other studies, topical administration of either 0.75% ropivacaine (6 mL) or 0.25% ropivacaine (5 mL) effectively reduces peri-extubation cough (18, 31). Since 1% ropivacaine is more accessible in clinical practice than 4% lidocaine, we chose ropivacaine topical anesthesia as a component of our multimodal intervention in the present study.

In our results, the addition of topical ropivacaine to dexmedetomidine-remifentanil combination reduced emergence cough by 29% and moderate-to-severe cough by 44% when compared with no intervention; the multimodal intervention also reduced emergence cough by 22% and moderate-to-severe cough by 44% when compared with the double intervention. For patients recovering from general anesthesia, emergence cough occurs mainly due to the direct stimulation of endotracheal tube on airway mucosa (32); topical anesthesia reduces cough reaction mainly by blocking afferent nociceptive stimuli (33). Our results are in line with above findings with topical ropivacaine anesthesia (18, 30, 31). Among our patients, the efficacy of multimodal prevention was less prominent in the subgroup of smokers, possibly due to the harmful effects of smoking on the airway mucosa (34).

Sore throat is one of the most common complaints in patients after general anesthesia with endotracheal tube, and is generally attributed to intubation-and extubation-related laryngeal injury (35). In the present study, patients given multimodal intervention experienced lighter sore throat than patients given no intervention. This indicates that, by reducing emergence cough, multimodal intervention may have reduced mucosa injury and therefore relieved sore throat. Previous studies reported that maintaining a low-dose remifentanil infusion, a single dose dexmedetomidine, or topical airway anesthesia each reduces hemodynamic changes during extubation (4, 36, 37). In our results, although multimodal intervention reduced emergence cough and moderate-to-severe cough, it did not significantly reduce hemodynamic fluctuation during the emergence period. This is possibly due to the small sample size. A larger trial may be helpful to answer the question.

There are some limitations. First, we only enrolled patients with ASA classes I and II. This lowered the research risk but also limits the generalizability of our results. It is clear that smooth emergence is more important for high-risk patients. Second, we adopted a low-dose combination in our double intervention group and therefore did not find a significant effect in reducing emergence cough. Further studies are required to explore the optimal dosing regimen for this combination. Third, we did not include a group with topical anesthesia alone, and cannot demonstrate if the effect of multimodal intervention is better than that of topical anesthesia alone. As the beneficial effect was seen in ropivacaine studies, comparing ropivacaine only can clarify the efficacy of ropivacaine in the multimodal combination. Fourth, despite multimodal intervention, the incidence of emergence cough remained high in our patients.

## Conclusion

For adult patients undergoing endonasal surgery, the multimodal intervention including ropivacaine topical anesthesia, dexmedetomidine administration, and remifentanil infusion significantly reduced emergence cough without delaying anesthesia emergence or increasing adverse events. Further studies are required to clarify the effect of ropivacaine and to determine the optimal dosing regimen in this multimodal intervention.

## Data availability statement

The raw data supporting the conclusions of this article will be made available by the authors, without undue reservation.

## Ethics statement

The studies involving humans were approved by Biomedical Research Ethics Committee of Peking University First Hospital. The studies were conducted in accordance with the local legislation and institutional requirements. The participants provided their written informed consent to participate in this study.

## Author contributions

JX: Formal analysis, Funding acquisition, Methodology, Project administration, Resources, Writing – original draft. PS: Investigation, Writing – original draft. J-HM: Formal analysis, Methodology, Software, Writing – original draft. D-XW: Formal analysis, Methodology, Supervision, Writing – review & editing.

## References

[1] Wong TH Weber G Abramowicz AE. Smooth extubation and smooth emergence techniques: a narrative review. Anesthesiol Res Pract. (2021) 2021:1–10. doi: 10.1155/2021/8883257 PMC7822686 33510786

[2] Kim JH Ham SY Kim DH Chang CH Lee JS. Efficacy of single-dose dexmedetomidine combined with low-dose remifentanil infusion for cough suppression compared to high-dose remifentanil infusion: a randomized, controlled, non-inferiority trial. Int J Med Sci. (2019) 16:376–83. doi: 10.7150/ijms.30227 30911271 PMC6428982

[3] Hu S Li Y Wang S Xu S Ju X Ma L. Effects of intravenous infusion of lidocaine and dexmedetomidine on inhibiting cough during the tracheal extubation period after thyroid surgery. BMC Anesthesiol. (2019) 19:66. doi: 10.1186/s12871-019-0739-1, PMID: 31054568 PMC6500031

[4] Lee JS Choi SH Kang YR Kim Y Shim YH. Efficacy of a single dose of dexmedetomidine for cough suppression during anesthetic emergence: a randomized controlled trial. Can J Anaesth. (2015) 62:392–8. doi: 10.1007/s12630-014-0295-6, PMID: 25523837

[5] Fedak PW Kolb E Borsato G Frohlich DE Kasatkin A Narine K. Kryptonite bone cement prevents pathologic sternal displacement. Ann Thorac Surg. (2010) 90:979–85. doi: 10.1016/j.athoracsur.2010.05.009 20732527

[6] Miller KA Harkin CP Bailey PL. Postoperative tracheal extubation. Anesth Analg. (1995) 80:149–72. doi: 10.1097/00000539-199501000-00025 7802273

[7] Hamilton FW Gregson FKA Arnold DT Sheikh S Ward K Brown J. Aerosol emission from the respiratory tract: an analysis of aerosol generation from oxygen delivery systems. Thorax. (2022) 77:276–82. doi: 10.1136/thoraxjnl-2021-217577, PMID: 34737195 PMC8867281

[8] Minogue SC Ralph J Lampa MJ. Laryngotracheal topicalization with lidocaine before intubation decreases the incidence of coughing on emergence from general anesthesia. Anesth Analg. (2004) 99:1253–7. doi: 10.1213/01.ANE.0000132779.27085.52 15385385

[9] Yang SS Wang NN Postonogova T Yang GJ McGillion M Beique F. Intravenous lidocaine to prevent postoperative airway complications in adults: a systematic review and meta-analysis. Br J Anaesth. (2020) 124:314–23. doi: 10.1016/j.bja.2019.11.033, PMID: 32000978

[10] Zhao G Yin X Li Y Shao J. Continuous postoperative infusion of remifentanil inhibits the stress responses to tracheal extubation of patients under general anesthesia. J Pain Res. (2017) 10:933–9. doi: 10.2147/JPR.S123423, PMID: 28458576 PMC5402994

[11] Weerink MAS Barends CRM Muskiet ERR Reyntjens KMEM Knotnerus FH Oostra M. Pharmacodynamic interaction of remifentanil and dexmedetomidine on depth of sedation and tolerance of laryngoscopy. Anesthesiology. (2019) 131:1004–17. doi: 10.1097/ALN.0000000000002882, PMID: 31425170

[12] Schulz KF Altman DG Moher D. CONSORT 2010 statement: updated guidelines for reporting parallel group randomised trials. BMJ. (2010) 340:c332. doi: 10.1136/bmj.c332, PMID: 20332509 PMC2844940

[13] Lee JH Koo BN Jeong JJ Kim HS Lee JR. Differential effects of lidocaine and remifentanil on response to the tracheal tube during emergence from general anaesthesia. Br J Anaesth. (2011) 106:410–5. doi: 10.1093/bja/aeq396 21205628

[14] Ely EW Truman B Shintani A Thomason JWW Wheeler AP Gordon S. Monitoring sedation status over time in ICU patients: reliability and validity of the Richmond Agitation-Sedation Scale (RASS). JAMA. (2003) 289:2983–91. doi: 10.1001/jama.289.22.2983 12799407

[15] Luthra A Prabhakar H Rath GP. Alleviating stress response to tracheal extubation in neurosurgical patients: a comparative study of two infusion doses of dexmedetomidine. J Neurosci Rural Pract. (2017) 8:S49–s56. doi: 10.4103/jnrp.jnrp_91_17 28936072 PMC5602261

[16] Perelló-Cerdà L Fàbregas N López AM Rios J Tercero J Carrero E. ProSeal laryngeal mask airway attenuates systemic and cerebral hemodynamic response during awakening of neurosurgical patients: a randomized clinical trial. J Neurosurg Anesthesiol. (2015) 27:194–202. doi: 10.1097/ANA.0000000000000108, PMID: 25121397

[17] D’Aragon F Beaudet N Gagnon V Martin R Sansoucy Y. The effects of lidocaine spray and intracuff alkalinized lidocaine on the occurrence of cough at extubation: a double-blind randomized controlled trial. Can J Anaesth. (2013) 60:370–6. doi: 10.1007/s12630-013-9896-8 23370978

[18] Fang P Zong Z Lu Y Han X Liu X. Effect of topical ropivacaine on the response to endotracheal tube during emergence from general anesthesia: a prospective randomized double-blind controlled study. BMC Anesthesiol. (2018) 18:134. doi: 10.1186/s12871-018-0601-x, PMID: 30261837 PMC6161381

[19] Baftiu N Krasniqi I Haxhirexha K Domi R. Survey about the extubation practice among anaesthesiologists in Kosovo. Open Access Maced J Med Sci. (2018) 6:350–4. doi: 10.3889/oamjms.2018.083, PMID: 29531602 PMC5839446

[20] Rassam S Sandbythomas M Vaughan RS Hall JE. Airway management before, during and after extubation: a survey of practice in the United Kingdom and Ireland. Anaesthesia. (2005) 60:995–1001. doi: 10.1111/j.1365-2044.2005.04235.x 16179045

[21] Hans P Marechal H Bonhomme V. Effect of propofol and sevoflurane on coughing in smokers and non-smokers awakening from general anaesthesia at the end of a cervical spine surgery. Br J Anaesth. (2008) 101:731–7. doi: 10.1093/bja/aen271, PMID: 18818191

[22] Tung A Fergusson NA Ng N Hu V Dormuth C Griesdale DEG. Medications to reduce emergence coughing after general anaesthesia with tracheal intubation: a systematic review and network meta-analysis. Br J Anaesth. (2020) 124:480–95. doi: 10.1016/j.bja.2019.12.041, PMID: 32098647

[23] Kim HY Kwak HJ Lee D Lee JH Min SK Kim JY. Comparison of remifentanil concentrations with and without dexmedetomidine for the prevention of emergence cough after nasal surgery: a randomized double-blinded trial. BMC Anesthesiol. (2021) 21:136. doi: 10.1186/s12871-021-01358-x, PMID: 33941098 PMC8094520

[24] Jun NH Lee JW Song JW Koh JC Park WS Shim YH. Optimal effect-site concentration of remifentanil for preventing cough during emergence from sevoflurane-remifentanil anaesthesia. Anaesthesia. (2010) 65:930–5. doi: 10.1111/j.1365-2044.2010.06450.x, PMID: 20645945

[25] Lam F Lin YC Tsai HC Chen TL Tam KW Chen CY. Effect of Intracuff lidocaine on postoperative sore throat and the emergence phenomenon: a systematic review and meta-analysis of randomized controlled trials. PLoS One. (2015) 10:e0136184. doi: 10.1371/journal.pone.0136184 26288276 PMC4544846

[26] Gladston DV Padmam S Amma RO Koshy RC Krishna KMJ Vijayan J. A randomized controlled trial to study the effect of intratracheal and intravenous lignocaine on airway and hemodynamic response during emergence and extubation following general anesthesia. North Clin Istanb. (2022) 9:323–30. doi: 10.14744/nci.2021.33407, PMID: 36276564 PMC9514071

[27] George SE Singh G Mathew BS Fleming D Korula G. Comparison of the effect of lignocaine instilled through the endotracheal tube and intravenous lignocaine on the extubation response in patients undergoing craniotomy with skull pins: a randomized double blind clinical trial. J Anaesthesiol Clin Pharmacol. (2013) 29:168–72. doi: 10.4103/0970-9185.111668, PMID: 23878435 PMC3713661

[28] Martini E Cavallini GM Campi L Lugli N Neri G Molinari P. Lidocaine versus ropivacaine for topical anesthesia in cataract surgery. J Cataract Refract Surg. (2002) 28:1018–22. doi: 10.1016/S0886-3350(01)01225-1, PMID: 12036647

[29] Caccavale A Romanazzi F Imparato M Negri A Porta A Ferentini F. Ropivacaine for topical anesthesia in pterygium surgery with fibrin glue for conjunctival autograft. Cornea. (2010) 29:375–6. doi: 10.1097/ICO.0b013e3181ba7061, PMID: 20164749

[30] Groeben H Grosswendt T Silvanus MT Pavlakovic G Peters J. Airway anesthesia alone does not explain attenuation of histamine-induced bronchospasm by local anesthetics: a comparison of lidocaine, ropivacaine, and dyclonine. Anesthesiology. (2001) 94:423–8. doi: 10.1097/00000542-200103000-00010, PMID: 11374600

[31] Thangavelu R Ventakesh RR Ravichandran K. Comparison of effect of airway nebulization with lignocaine 2% versus ropivacaine 0.25% on intubation and extubation response in patients undergoing surgery under general anesthesia: a randomized double-blind clinical trial. Anesth Essays Res. (2018) 12:338–43. doi: 10.4103/aer.AER_83_18 29962594 PMC6020607

[32] Tanoubi I Sun JN Drolet P Fortier LP Donati F. Replacing a double-lumen tube with a single-lumen tube or a laryngeal mask airway device to reduce coughing at emergence after thoracic surgery: a randomized controlled single-blind trial. Can J Anaesth. (2015) 62:988–95. doi: 10.1007/s12630-015-0403-2 25985845

[33] Jee D Park SY. Lidocaine sprayed down the endotracheal tube attenuates the airway-circulatory reflexes by local anesthesia during emergence and extubation. Anesth Analg. (2003) 96:293–7. doi: 10.1213/00000539-200301000-00058, PMID: 12505969

[34] Kim V Jeong S Zhao H Kesimer M Boucher RC Wells JM. Current smoking with or without chronic bronchitis is independently associated with goblet cell hyperplasia in healthy smokers and COPD subjects. Sci Rep. (2020) 10:20133. doi: 10.1038/s41598-020-77229-1, PMID: 33208859 PMC7674445

[35] Brodsky MB Akst LM Jedlanek E Pandian V Blackford B Price C. Laryngeal injury and upper airway symptoms after endotracheal intubation during surgery: a systematic review and meta-analysis. Anesth Analg. (2021) 132:1023–32. doi: 10.1213/ANE.0000000000005276, PMID: 33196479 PMC7969363

[36] Nho JS Lee SY Kang JM Kim MC Choi YK Shin OY. Effects of maintaining a remifentanil infusion on the recovery profiles during emergence from anaesthesia and tracheal extubation. Br J Anaesth. (2009) 103:817–21. doi: 10.1093/bja/aep307, PMID: 19864308

[37] Meng YF Cui GX Gao W Li ZW. Local airway anesthesia attenuates hemodynamic responses to intubation and extubation in hypertensive surgical patients. Med Sci Monit. (2014) 20:1518–24. doi: 10.12659/MSM.890703 25175842 PMC4156342

