# Supplementary material for: Multimodal prevention of emergence cough following nasal endoscopic surgery under general anesthesia: a double-blind randomized trial
Source: Front Med (Lausanne). 2024 Jan 24;11:1288978. doi: 10.3389/fmed.2024.1288978 (PMC10847355; doi:10.3389/fmed.2024.1288978)
Supplement: Supplementary file 1 [file Table_1.DOCX]

**Supplement Table S1.** Grade of emergence cough and Richmond Agitation-Sedation Scale after surgery.

|  | Control  (*N* = 50) | Double intervention  (*N* = 50) | Multimodal intervention  (*N* = 50) | Multimodal *vs.* control | | Multimodal *vs.* double | | Double *vs.* control | |
| --- | --- | --- | --- | --- | --- | --- | --- | --- | --- |
|  |  |  |  | MD (95% CI) | *P* value ^†^ | MD (95% CI) | *P* value ^†^ | MD (95% CI) | *P* value ^†^ |
| ***Emergence cough* ^‡^** |  |  |  |  |  |  |  |  |  |
| Overall^§^ |  |  |  |  | **<0.001**^b^ |  | **<0.001**^b^ |  | 0.08 ^c^ |
| None ^ǂ^ | 1 (2%) | 5 (10%) | 15 (30%) | --- |  | --- |  | --- |  |
| Mild ^※^ | 6 (12%) | 2 (4%) | 11 (22%) | --- |  | --- |  | --- |  |
| Moderate ^††^ | 16 (32%) | 23 (46%) | 17 (34%) | --- |  | --- |  | --- |  |
| Severe ^‡‡^ | 27 (54%) | 20 (40%) | 7 (14%) | --- |  | --- |  | --- |  |
| Overall ^c^ | 3 [2, 3] | 2 [2, 3] | 0 [1, 2] | -1 (-1, -1) | **<0.001** ^a^ | -1 (-1, 0) | **<0.001** ^a^ | 0 (0, 0) | 0.22 ^a^ |
| Pre-extubation |  |  |  |  | **<0.001** ^c^ |  | **<0.001** ^c^ |  | 0.09 ^b^ |
| None ^ǂ^ | 7 (14%) | 13 (26%) | 30 (60%) | --- |  | --- |  | --- |  |
| Mild ^※^ | 5 (10%) | 8 (16%) | 7 (14%) | --- |  | --- |  | --- |  |
| Moderate ^††^ | 17 (34%) | 19 (38%) | 13 (26%) | --- |  | --- |  | --- |  |
| Severe ^‡‡^ | 21(42%) | 10 (20%) | 0 (0%) | --- |  | --- |  | --- |  |
| Pre-extubation | 2 [2, 3] | 2 [0, 2] | 0 [0, 2] | -2 (-2, -1) | **<0.001** ^a^ | -1 (-1, 0) | **<0.001** ^a^ | -1 (-1, 0) | **0.01** ^a^ |
| Upon extubation |  |  |  |  | **<0.001** ^b^ |  | **<0.001** ^b^ |  | 0.03^c^ |
| None ^ǂ^ | 1 (2%) | 6 (12%) | 18 (36%) | --- |  | --- |  | --- |  |
| Mild ^※^ | 7 (14%) | 4 (8%) | 14 (28%) | --- |  | --- |  | --- |  |
| Moderate ^††^ | 17 (34%) | 26 (52%) | 11 (22%) | --- |  | --- |  | --- |  |
| Severe ^‡‡^ | 25 (50%) | 14 (28%) | 7 (14%) | --- |  | --- |  | --- |  |
| Upon extubation | 3 [2, 3] | 2 [2, 3] | 1 [0, 2] | -1 (-2, -1) | **<0.001** ^a^ | -1 (-1, 0) | **<0.001** ^a^ | 0 (-1, 0) | 0.04 ^a^ |
| Post-extubation |  |  |  |  | 0.05 ^c^ |  | 0.46 ^c^ |  | 0.36 ^c^ |
| None ^ǂ^ | 31 (62%) | 37 (74%) | 42 (84%) | --- |  | --- |  | --- |  |
| Mild ^※^ | 11 (22%) | 6 (12%) | 4 (8%) | --- |  | --- |  | --- |  |
| Moderate^††^ | 7 (14%) | 7 (14%) | 4 (8%) | --- |  | --- |  | --- |  |
| Severe ^‡‡^ | 1 (2%) | 0 (0%) | 0 (0%) | --- |  | --- |  | --- |  |
| Post-extubation | 0 [0, 1] | 0 [0, 1] | 0 [0, 0] | 0 (0, 0) | **0.016** ^a^ | 0 (0, 0) | 0.22 ^a^ | 0 (0, 0) | 0.25 ^a^ |
| ***RASS after extubation*** |  |  |  |  |  |  |  |  |  |
| At 5 min |  |  |  |  | 0.46 ^c^ |  | 0.34 ^c^ |  | 0.15 ^c^ |
| -2 | 11 (22%) | 18 (36%) | 12 (24%) | --- |  | --- |  | --- |  |
| -1 | 29 (58%) | 29 (58%) | 33 (66%) | --- |  | --- |  | --- |  |
| 0 | 8 (16%) | 3 (6%) | 3 (6%) | --- |  | --- |  | --- |  |
| 1 | 1 (2%) | 0 (0%) | 2 (4%) | --- |  | --- |  | --- |  |
| 2 | 1 (2%) | 0 (0%) | 0 (0%) | --- |  | --- |  | --- |  |
| At 5 min | -1 [-1, -1] | -1 [-2, -1] | -1 [-1, -1] | 0 (0, 0) | 0.37 ^a^ | 0 (0, 0) | 0.16 ^a^ | 0 (0, 0) | 0.03 ^a^ |
| At 20 min |  |  |  |  | >0.99 ^c^ |  | 0.49 ^c^ |  | 0.73 ^c^ |
| -2 | 1 (2%) | 2 (4%) | 0 (0%) | --- |  | --- |  | --- |  |
| -1 | 11 (22%) | 13 (26%) | 12 (24%) | --- |  | --- |  | --- |  |
| 0 | 38 (76%) | 35 (70%) | 38 (76%) | --- |  | --- |  | --- |  |
| At 20 min | 0 [0, 0] | 0 [-1, 0] | 0 [0, 0] | 0 (0, 0) | 0.96 ^a^ | 0 (0, 0) | 0.44 ^a^ | 0 (0, 0) | 0.48 ^a^ |

Data are *n* (%) or median [interquartile range]. MD, median difference; CI, confidence interval.

^†^ *P* values <0.017 are considered statistically significant after Bonferroni corrections.

^‡^ The severity of cough was classified into four grades: grade 0, no cough; grade 1, single cough; grade 2, unsustained (<5 s) cough; grade 3, sustained (>5 s) cough or bucking.

^§^Patients who developed cough during the period from the end of surgery to five minutes after extubation.

^ǂ^ None cough episode.

^※^ Single cough.

^††^ More than one episode of unsustained (<5 seconds) coughing.

^‡‡^ Sustained (>5 seconds) bout(s) of coughing.

^a^ Kruskal-Wallis test.

^b^ Chi-squared test.

^c^ Fisher’s exact test.
